# Impact of Procedural Sedation on the Clinical Outcome of Microelectrode Recording Guided Deep Brain Stimulation in Patients with Parkinson’s Disease

**DOI:** 10.3390/jcm10081557

**Published:** 2021-04-07

**Authors:** Michael J. Bos, Dianne de Korte-de Boer, Ana Maria Alzate Sanchez, Annelien Duits, Linda Ackermans, Yasin Temel, Anthony R. Absalom, Wolfgang F. Buhre, Mark J. Roberts, Marcus L. F. Janssen

**Affiliations:** 1 Department of Anesthesiology and Pain Medicine, Maastricht University Medical Center, P. Debyelaan 25, 6229 HX Maastricht, The Netherlands; dianne.de.korte@mumc.nl (D.d.K.-d.B.); wolfgang.buhre@mumc.nl (W.F.B.); 2 Faculty of Health, Medicine and Life Sciences, School for Mental Health and Neuroscience, Maastricht University, Universiteitssingel 40, 6229 ER Maastricht, The Netherlands; ana.alzatesanchez@mumc.nl (A.M.A.S.); annelien.duits@maastrichtuniversity.nl (A.D.); y.temel@maastrichtuniversity.nl (Y.T.); m.janssen@maastrichtuniversity.nl (M.L.F.J.); 3 Department of Medical Psychology, Maastricht University Medical Center, P. Debyelaan 25, 6229 HX Maastricht, The Netherlands; 4 Department of Neurosurgery, Maastricht University Medical Center, P. Debyelaan 25, 6229 HX Maastricht, The Netherlands; linda.ackermans@mumc.nl; 5 Department of Anesthesiology, University Medical Center Groningen, Groningen University, Hanzeplein 1, 9713 GZ Groningen, The Netherlands; a.r.absalom@umcg.nl; 6 Faculty of Psychology and Neuroscience, Maastricht University, Universiteitssingel 40, 6229 ER Maastricht, The Netherlands; mark.roberts@maastrichtuniversity.nl; 7 Department of Clinical Neurophysiology, Maastricht University Medical Center, P. Debyelaan 25, 6229 HX Maastricht, The Netherlands

**Keywords:** deep brain stimulation, levodopa equivalent daily dosage, local anesthesia, microelectrode recordings, MDS-UPDRS III, subthalamic nucleus, Parkinson’s disease, procedural sedation and/or analgesia

## Abstract

Background: Subthalamic nucleus (STN) deep brain stimulation (DBS) has become a routine treatment of advanced Parkinson’s disease (PD). DBS surgery is commonly performed under local anesthesia (LA) to obtain reliable microelectrode recordings. However, procedural sedation and/or analgesia (PSA) is often desirable to improve patient comfort. The impact of PSA in addition to LA on outcome is largely unknown. Therefore, we performed an observational study to assess the effect of PSA compared to LA alone during STN DBS surgery on outcome in PD patients. Methods: Seventy PD patients (22 under LA, 48 under LA + PSA) scheduled for STN DBS implantation were included. Dexmedetomidine, clonidine or remifentanil were used for PSA. The primary outcome was the change in Movement Disorders Society Unified Parkinson’s Disease Rating Score III (MDS-UPDRS III) and levodopa equivalent daily dosage (LEDD) between baseline, one month before surgery, and twelve months postoperatively. Secondary outcome measures were motor function during activities of daily living (MDS-UPDRS II), cognitive alterations and surgical adverse events. Postoperative assessment was conducted in “on” stimulation and “on” medication conditions. Results: At twelve months follow-up, UPDRS III and UPDRS II scores in “on” medication conditions were similar between the LA and PSA groups. The two groups showed a similar LEDD reduction and an equivalent decline in executive function measured by the Stroop Color-Word Test, Trail Making Test-B, and verbal fluency. The incidence of perioperative and postoperative adverse events was similar between groups. Conclusion: This study demonstrates that PSA during STN DBS implantation surgery in PD patients was not associated with differences in motor and non-motor outcome after twelve months compared with LA only.

## 1. Introduction

Deep brain stimulation (DBS) of the subthalamic nucleus (STN) is an effective treatment for patients with advanced Parkinson’s disease (PD). It improves motor and non-motor symptoms and quality of life and alleviates levodopa-related side effects in patients with advanced PD [1,2,3]. The surgical procedure involves the implantation of electrodes into the sensorimotor region of the STN for the delivery of electrical current generated by an implanted pulse generator. Precise electrode placement is the key component during DBS surgery and is of paramount importance for postoperative prognosis [4]. Several techniques are used to achieve accurate target localization and electrode placement. First, high-resolution neuroimaging techniques using MRI are applied to identify the STN for stereotactic targeting. Second, microelectrode recording (MER) is often used to pinpoint the dorsal, lateral and ventral boundaries of the STN according to the neurophysiological signature [5]. Finally, intraoperative macrostimulation can be used to assess the therapeutic window [6,7,8]. There is as of yet no consensus on the use of techniques, and the choice depends on the experience and preference of the local DBS surgical team.

The anesthetic management techniques applied are local anesthesia (LA), procedural sedation and/or analgesia (PSA) or general anesthesia. There is as of yet no consensus on which approach should be used [9].

Many centers employing MER-based DBS surgery prefer to perform the procedure under awake conditions to obtain reliable neurophysiological mapping and enable intraoperative test stimulation to observe the motor improvement and potential side-effects. In our recent work we have shown that anesthetic agents alter the firing characteristics of the STN, which is consistent with data obtained from previous studies [10,11,12,13,14,15]. These findings provide important arguments to avoid the use of anesthetic drugs during DBS surgery. However, patients may experience stress due to “off”-period symptoms, anxiety, or pain during the procedure [16]. To improve patient comfort and tolerance, PSA or general anesthesia can be applied. An important advantage of PSA over general anesthesia is that communication with the patient remains possible allowing clinical testing. Furthermore, PSA entails lower anesthetic drug doses and consequently less profound effects on MER.

In the last decade, several clinical reports and reviews have been published with satisfying clinical results when DBS surgery was performed under sedation or general anesthesia [17,18,19,20]. However, it remains difficult to interpret these findings due to heterogeneity in DBS targets, surgical (intraoperative neuroimaging versus MER), and anesthesia techniques (awake versus sedation versus general anesthesia). While a number of studies have reported minor effects of PSA on MER, it remains unclear whether these effects impact clinical outcome measures [21,22,23].

We therefore performed a retrospective observational cohort study. The primary aim of this study was to assess the effect of PSA in addition to LA compared to LA alone, during MER-based STN DBS electrode implantation in PD patients on motor outcome. We also assessed the effect on neuropsychological outcome parameters and activities of daily living in both groups.

## 2. Materials and Methods

### 2.1. Study Design

Data were retrospectively collected from the records of patients with PD who underwent bilateral STN DBS electrode implantation surgery at the Maastricht University Medical Center from May 2000 to December 2018. All patients who received LA alone or PSA with LA and had intraoperative MER for target localization were selected. Other inclusion criteria were availability of surgical, anesthetic, and neuropsychological records. Patients who underwent DBS surgery under general anesthesia were excluded. We screened 93 consecutive PD patients from our database and found 70 patients who met the inclusion and exclusion criteria. Patients were divided into two groups: LA and PSA with LA. The study was approved by the local ethical committee (METC Maastricht University Medical Center, the Netherlands, protocol number 184214).

### 2.2. Motor Evaluation

The primary outcome of this study was the change from baseline in the Movement Disorders Society Unified Parkinson’s Disease Rating Score III (MDS-UPDRS III) score at 12 months. Briefly, the MDS-UPDRS is a four-part measurement instrument to quantify the motor and non-motor impairment and disability of PD patients. Part III scores are used for motor examination. Higher scores for each of the MDS-UPDRS subscores indicate a more severe impairment [24]. Patients were assessed one month before surgery and twelve months postoperatively. Motor function during activities of daily living was assessed with the MDS-UPDRS II score, and the level of disability was assessed with the Hoehn and Yahr score [25]. Due to a revision of UPDRS into MDS-UPDRS scores from 2008, all UPDRS scores were recalculated to MDS-UPDRS scores to create uniformity [26]. Preoperatively, patient motor function scores were evaluated in “on” medication and compared to motor function scores in “on” medication and “on” stimulation after twelve months. In addition to the functional scores, we measured changes in levodopa dose-equivalent (LEDD) at baseline and at twelve months post-surgery [27].

### 2.3. Neuropsychological Outcome

A neuropsychologist conducted the neuropsychological tests within one month before surgery and again twelve months after surgery. The neuropsychological evaluation included the Rey Auditory Verbal Learning Test (RAVLT) to assess verbal learning and memory function, the Stroop Colour-Word Test to measure mental speed and selective attention, verbal fluency to measure semantic (category) and phonic (letter) fluency, and the Trail Making Test (TMT) part B to assess cognitive flexibility [28,29,30,31].

### 2.4. Surgical Technique

In all patients, the surgical technique involved two phases: implantation of the probes and implantation of the pulse generator. The first stage was performed under LA alone or LA together with PSA. A Leksell stereotactic frame (Elekta, Stockholm, Sweden) was placed, and computed tomography (CT) imaging of the head was performed. The CT-image was co-registered with a magnetic resonance imaging (MRI) image that had been acquired the day prior to surgery. From 2000 to 2012, atlas-based standard coordinates (indirect targeting) were used. With the advances in MR technology, we changed our approach to direct targeting using T2 weighted MR imaging (1.5 or 3 T). Trajectory planning was performed on contrast-enhanced T1 weighted MR imaging with a 1 mm slice thickness. The STN targets were identified on the images, and the corresponding frame-based coordinates and trajectories were calculated. Additional local anesthetic infiltration was administered prior to skin incision and drilling of burr holes. Microelectrodes (model 230766, Medizintechnik GmbH, Emmendingen, Germany) were inserted and recordings were performed. Electrophysiological data were used to judge the depths of the dorsal and ventral borders of the STN. Macrostimulation was used to assess the therapeutic effect and stimulation-related side effects. The choice of final electrode location was based on the presence of adequate electrophysiological recordings and test stimulation. Before fixation of the final electrodes, fluoroscopy was used to confirm the position. The same procedure was performed on the contralateral side. After positioning of the electrodes, the second phase was performed under general anesthesia. A pulse generator was implanted (either infraclavicularly or on the abdominal wall) and connected to the DBS electrodes with tunneled wires. Within 24 h after surgery, the patients underwent a CT or MRI scan to evaluate the final lead position and exclude any surgery-related complications.

### 2.5. MER Acquisition

MER was performed with up to five concentrically arranged microelectrodes to record neuronal activity along the planned trajectory to identify the sensorimotor region of the STN. The MER track started 10 mm above the presumed target and MER were performed at steps of 0.5–1 mm up to 5–7 mm below target. MER was sampled for around 30 s at each depth. We defined the position of the dorsal border of the STN as the point at which a sudden increase in baseline activity and increase in discharge rate with a rhythmic burst activity was detected [10]. The electrode signals were sampled at 20 or 25 kHz, bandpass-filtered online (V3.15; Inomed Medizintechnik GnmH, Emmendingen, Germany), and saved for offline analysis. From these recordings, the average and maximum length (distance from dorsal to ventral border) of the STN was determined.

### 2.6. Anesthetic Management

All patients underwent a preoperative assessment of eligibility for DBS surgery by an anesthesiologist. In the operation room, standard monitoring was applied including pulse oximetry, inspiratory and expiratory O_2,_ and CO_2_ monitoring, a five-lead electrocardiogram, and non-invasive and/or invasive blood pressure monitoring. Supplemental oxygen was administered by a nasal cannula. The stereotactic pin sites and surgical incision sites were infiltrated with a 50:50 mixture of lidocaine 1% and levobupivacaine 0.5% with epinephrine (1:100,000). DBS surgery was performed under LA alone or with additional PSA according to the preference of the anesthesiologist. For PSA, the anesthesiologist administered either dexmedetomidine (by intravenous infusion at a rate of 0.2–0.6 µg/kg/min), or clonidine (as an intravenous bolus of 75–225 µg, followed by continuous infusion of 20 µg/h) or remifentanil (by continuous infusion of 0.02–0.06 µg/kg/min^/^) titrated until the patient was comfortable. PSA was continued during MER and up to the end of the first stage. In the second stage, during implantation of the pulse generator, all patients received general anesthesia, with the choice of anesthetic agents at the discretion of the responsible anesthesiologist. Postoperatively, patients were cared for on a post-anesthesia care unit for 24 h.

### 2.7. Adverse Events

Adverse events were identified and divided into perioperative adverse events and late adverse events (>2 weeks of surgery up to twelve months).

### 2.8. Statistical Analysis

Baseline characteristics were summarized as the mean and standard deviation for continuous data and number with percentage for nominal data. The equivalence of the baseline characteristics of the LA and the PSA group was evaluated with a two-sample *t*-test or χ^2^ test.

To test for group differences in the primary outcome, change in MDS-UPDRS III scores (i.e., 12 months score minus baseline score), and the secondary motor outcomes, change in LEDD and MDS-UPDRS II scores, univariate and multivariate linear regression models were used. All multivariate models were adjusted for age, sex, and disease duration. The MDS-UPDRS III model was additionally adjusted for UPDRS-III at baseline in “off” medication conditions and LEDD at 12 months, and the MDS-UPDRS II model for UPDRS-II at baseline in “off” medication conditions.

Preoperative neuropsychological variables were analyzed with independent-samples *t*-tests to assess between-group differences with corrections applied as appropriate with Levene’s test. To compare the difference in neuropsychological change between the LA and PSA group, pre- and postoperatively, we used the Mann–Whitney U test based on change scores.

To analyze the effect of PSA MER-based mapping of the STN, we performed for STN length (average and maximum) a two-sample *t*-test. A multiple regression analysis was performed to examine the association between PSA and demographic variables on STN average and maximum length. The significance level was set at *p* < 0.05 for all analyses. The analysis of MER data was performed using MATLAB scripts (V2017B; Mathworks, Natick, MA, USA). The statistical analysis of motor and neuropsychological outcomes were conducted with SPSS version 26.

## 3. Results

### 3.1. Baseline Patient Characteristics

Seventy patients who underwent bilateral STN DBS surgery were included in the study. Twenty-two received LA only (the “LA group”) and 48 patients received LA and PSA (“PSA group”). In the PSA group, 9 patients received dexmedetomidine, 3 patients dexmedetomidine and remifentanil, 15 patients clonidine, 12 patients clonidine and remifentanil, and 9 patients remifentanil. Demographic data are summarized in Table 1.

### 3.2. Motor Outcome and Activities of Daily Living

Motor score changes scores are provided in Table 2. From univariate and multiple linear regression models, we observed no difference in MDS-UPDRS III change scores during “on”-medication and “on”-stimulation conditions at 12 months between groups (*p* = 0.67). Furthermore, MDS-UPDRS II change scores showed no difference between groups after 12 months (*p* = 0.87). The mean LEDD reduction in the LA group was similar to that in the PSA group (LA: −622 ± 560 mg (42.9%), PSA: −674 ± 559 (52.5%); *p* = 0.59) at 12 months.

### 3.3. Neuropsychological Outcome

The neuropsychological test scores at baseline and after 12 months and the change scores are summarized in Table 3. The Levene’s test showed that the neuropsychological profiles of the LA and PSA group at baseline were similar, with exception of the Stroop Colour-Word 3 test (LA 103.8 ± 20.7 vs. PSA 114.3 ± 50.6 *p* = 0.035). The neuropsychological change scores after 12 months showed a significant decline in executive function, verbal fluency (category and letters) and the Stroop Colour-Word Test card 3 and TMT-B). This decline was not different between the LA and PSA groups. No decline was noted on verbal memory measures (AVLT learning and AVLT memory) in both groups.

### 3.4. STN Borders and Pass Rate

MER data could be retrieved from 58 patients. Data were acquired from 30 hemispheres in the LA group and 86 hemispheres in the PSA group. The pass rate through the STN was similar in both groups (LA 0.78 ± 0.2, PSA 0.79 ± 0.2 (t(114) = −0.12, *p* = 0.91)). Furthermore, there were no differences in the longest STN trajectory (LA 5.8 ± 1.4 mm versus PSA 5.6 ± 1.4 mm (t(114) = 0.94, *p* = 0.35)) and average length of the electrodes through the STN between the two groups (LA 4.7 ± 1.2 mm versus PSA 4.5 ± 1.2 mm (t(114) = 0.98, *p* = 0.33)).

### 3.5. Adverse Events

The most common adverse event was hypertension (defined as a systolic blood pressure of >160 mmHg). Hypertension was treated with nicardipine, hydralazine, or labetalol. One patient in the LA group suffered a pulmonary embolism, as a result of which the procedure had to be aborted. One patient in the PSA group had an intraoperative seizure, which was effectively treated with anti-epileptic medication allowing the procedure to be completed. Two patients (1 in the LA and 1 in the PSA group) developed postoperative delirium. Intracranial hemorrhage was not observed. Late adverse events consisted of wound infections requiring pulse generator or lead removal (three patients in the LA group and three in the PSA group) and hardware related complications such as lead fracture or high impedance (two patients in the LA group).

## 4. Discussion

In this retrospective study, we found no evidence that PSA during MER-guided STN DBS electrode implantation influences the clinical outcome after one year in patients with PD. The motor outcome, degree of LEDD reduction, neuropsychological outcome, and activities in daily life were the same in both groups. In line with these findings, we showed that PSA did not adversely affect the MER-based assessments of STN length.

The optimal anesthetic management regimen during DBS surgery is still debated in the current literature. Proponents of DBS under LA alone emphasize that anesthetic agents alter MER, thereby potentially interfering with the optimal placement of the electrode in the target area. Additionally, intraoperative testing is regarded by some to result in fewer postoperative treatment-induced side-effects and a longer duration of DBS efficacy [7,19]. However, patients having their procedure performed under LA only may suffer from restlessness, fatigue, anxiety, agitation, or severe “off” symptoms. The possibility of undergoing DBS surgery under PSA may improve patient acceptance of the procedure and therefore increase the number of patients who can be treated. Adequately performed PSA can create patient comfort combined with cooperation allowing intraoperative testing.

Historically, propofol has been widely used for PSA in surgical procedures. However, propofol easily impairs consciousness and can alter stimulation thresholds and interfere with MER, even in low doses, making it a sub-optimal choice for DBS surgery [32,33]. In our daily practice, when PSA is required, we use the α_2_-agonists dexmedetomidine or clonidine, and in some cases either remifentanil alone or in addition to α_2_-agonists. Dexmedetomidine and clonidine produce dose-dependent sedation, analgesia, and anxiolysis [33,34]. Contrary to most sedatives, α_2_ agonists do not have a direct gamma-aminobutyric acid (GABA)-ergic effect and so have a less profound effect on MER compared to the GABA-ergic drugs [10,32]. Remifentanil, acting mainly on the mu opioid receptor agonist, has limited effects on STN neuronal activity at low doses, though a recently published report showed a significant reduction in STN neuronal activity with doses above 0.1 µg/kg/min [10,15].

### 4.1. Influence of PSA on Motor Outcome

To the best of our knowledge, our study is the first to describe and compare the use of non-GABA-ergic PSA on motor outcome with a control group who only (preop) received LA during bilateral MER-based STN DBS surgery. Only two studies previously addressed the effect of dexmedetomidine on outcome. One single cohort study evaluated outcomes in a group of 48 PD patients who received a dexmedetomidine-based regimen during STN DBS surgery with the outcomes of a group of 20 patients who received a remifentanil-based regimen. They found reduced UPDRS III scores of 18.2% (stimulation “on”, medication “off”) and a LEDD reduction of almost 52% at 6 months in the dexmedetomidine group. In the remifentanil group a reduction in UPDRS III of 16.3% (stimulation “on”, mediation “off”) and a LEDD reduction of 45.7% was observed after 6 months. Of note, sedatives were discontinued 20–30 min before MER, and patients in both groups also received midazolam. There were no significant differences in motor symptoms improvement or LEDD reduction among the sedative regimens [21]. In another single cohort study, Morace et al. investigated the effect of continuous low-dose dexmedetomidine infusion (range 0.3–0.5 µg/kg/h) during unilateral STN DBS surgery in 11 PD patients. Their findings showed a mean reduction in UPDRS III scores of 39% (stimulation “on”, medication “off”) and a mean reduction in LEDD of 45.9% after 12 months [35]. It is difficult to compare the data of motor scores from these studies with the data from our study, as we determined MDS-UPDRS III scores in stimulation “on” and medication “on” conditions; however, it is noteworthy that the reduction in LEDD after 12 months that we found is comparable to that of other studies. The effect of clonidine or remifentanil, as part of PSA, on motor outcomes has not been evaluated before.

### 4.2. Influence of PSA on Neuropsychological Outcome

In line with the available literature, we observed a significant decline in executive function after 12 months, independent of the anesthetic technique [36]. Overall, our results show that DBS in PD patients affects some domains of cognition, but these changes are independent of the anesthetic technique. Although the beneficial effects of STN DBS surgery on motor outcome and quality of life are well recognized, its effect on neuropsychological function has shown heterogeneous results. These variable results are most likely attributed to inhomogeneity in neuropsychological tests, population characteristics, and surgical techniques [37,38].

### 4.3. Influence of PSA on Target Identification

Precise placement of the DBS lead into the sensori-motor region of the STN is required to ensure the efficacy of the treatment and to minimize adverse effects [39]. Intraoperative localization of the dorsolateral STN can be achieved by MER. In a previous study, we examined the effect of PSA with dexmedetomidine, clonidine, and remifentanil on neuronal activity during STN DBS [10]. We observed a dose-dependent decrease in multi-unit activity associated with dexmedetomidine. Other research groups found similar dose-dependent effects of dexmedetomidine on STN neuronal activity [11,40]. These mild effects on neuronal activity appear to have no negative impact on the ability to identify the STN since we and two previous case series found that dexmedetomidine had no effect on STN border identification or length based on MER [35,41].

The effect of remifentanil on STN neural activity was examined in two previous studies. In one, four PD patients received a single bolus of remifentanil (0.05 mg/kg), which had little effect on STN activity [12]. In the second, more recent study, the effect of continuous infusion of remifentanil (0.1 µg/kg/min) on neuronal activity was investigated. The authors found that remifentanil interferes with the dorsolateral identification of the STN [15]. Unfortunately, the positioning accuracy of the electrode implantation was not reported, and therefore the clinical relevance of these findings remain unclear. Both studies are difficult to compare with the present study because of differences in sedation regimes. However, our findings suggest that remifentanil seems to have minimal relevant effects on target identification when low dosages are used (<0.06 µg/kg/min).

The effect of clonidine on STN identification has not been studied before. Clonidine might modulate the noradrenergic input from the locus coeruleus to the STN [42]. In our previous study, we were not able to observe the effects of clonidine on MER. In line with these findings, in the present study, clonidine showed no relevant effects on STN identification.

### 4.4. Limitations

The present study has some important limitations. First, this was a retrospective analysis over a long period of time. As a consequence, there are fewer patients in the LA group and outcome data are incomplete. Secondly, the anesthetic technique was not pre-specified. Assignment to one of the groups was not at random but depended on the clinical observations and judgment of the anesthesiologist meaning that patients with severe “off” medication symptoms or other stress-related symptoms may have been more likely to receive PSA. Although our analysis of demographic variables between groups suggested no such systematic difference, such a confound remains a concern. Thirdly, we did not use processed electroencephalography-based depth of hypnosis/anesthesia monitoring (e.g., with a bispectral index monitor (Medtronic, Dublin, Ireland) or state/response entropy monitoring (GE Healthcare, Helsinki, Finland)), and neither did we perform clinical assessments of the depth of sedation. We are therefore unable to rule out the possibility that DBS outcomes may have been impacted in more heavily sedated patients. Finally, we used a variety of anesthetic agents, leading to small group sizes, and so we were not able to perform subgroup analysis. Therefore, we can only draw cautious conclusions based on the combined results of the whole PSA group.

Although the study population was small, this is the largest retrospective analysis of data from PD patients in whom sedatives were administered during DBS surgery, including during MER to date.

## 5. Conclusions

The clinical success of STN DBS depends partly on accurate placement of the DBS electrode. MER and test stimulation in awake (unsedated) patients has long been considered as the golden standard for this procedure. This study indicates that PSA in addition to LA has no negative impact on clinical outcome.

## Figures and Tables

**Table 1:** Baseline patient characteristics: local anesthesia versus procedural anesthesia and/or analgesia.

	LA (*n* = 22)	PSA (*n* = 48)	*p*-Value
Age at DBS (years)	59 ± 8.8	60 ± 8.0	0.63
Gender (female)	9 (41%)	20 (42%)	0.95
Disease duration (months)	123 ± 47	119 ± 52	0.76
MDS-UPDRS II pre-op (“off”)	15.9 ± 6.5	16.3 ± 4.6	0.81
MDS-UPDRS II pre-op (“on”)	9.4 ± 5.3	9.5 ± 4.9	0.93
MDS-UPDRS III pre-op (“off”)	47.8 ± 18.3	44.8 ± 12.1	0.43
MDS-UPDRS III pre-op (“on”)	20.7 ± 10.5	22.7 ± 11.8	0.52
H&Y (“off”)	2.9 ± 0.8	2.8 ± 0.7	0.52
H&Y (“on”)	2.5 ± 0.3	2.4 ± 0.5	0.40
LEDD (mg/day)	1451 ± 788	1284 ± 619	0.34

Results are presented as means (SD), or percentage. DBS: deep brain stimulation, H&Y: Hoehn and Yahr Scale, LEDD: Levodopa equivalent daily dosage, LA: local anesthesia, MDS-UPDRS: Movement Disorders Society Unified Parkinson’s Disease Rating Scale, PSA: procedural anesthesia and/or analgesia.

**Table 2:** Motor outcome change scores at 12 months.

	*n*	Baseline	12 Months	Change Score	*p*-Value
MDS-UPDRS III (“on”)					
LA	20	20.7 ± 10.5	21.8 ± 13.6	1.07 ± 15.2	0.67
PSA	43	22.7 ± 11.8	24.6 ± 13.2	1.85 ± 13.4	
MDS-UPDRS II (“on”)					
LA	14	9.4 ± 5.3	10.5 ± 4.9	1.67 ± 5.1	0.87
PSA	28	9.5 ± 4.9	11.8 ± 6.4	2.23 ± 5.6	
LEDD					
LA	22	1451 ± 788	829 ± 712	−622 ± 560	0.59
PSA	48	1284 ± 619	626 ± 376	−674 ± 559	

*p*-value for group difference in change score from multivariate linear regression models; all models are adjusted for age, sex, and disease duration. The MDS-UPDRS III model was additionally adjusted for UPDRS-III at baseline “off” and LEDD at 12 months, and the MDS-UPDRS II model for UPDRS-II at baseline “off”. MDS-UPDRS: Movement Disorders Society Unified Parkinson’s Disease Rating Scale, LA: local anesthesia, LEDD: Levodopa equivalent daily dosage, PSA: Procedural sedation and/or analgesia.

**Table 3:** Neuropsychological test scores at baseline and after 12 months.

TEST	*n*	Baseline	12 Months	Change Score	*p*-Value
Stroop Color-Word test					
LA	21	103.76 ± 20.70	119.20 ± 21.77	15.40 ± 15.45	0.307
PSA	47	114.26 ± 50.53	142.83 ± 85.36	28.57 ± 67.09	
Fluency category					
LA	21	41.05 ± 8.01	36.71 ± 11.21	−4.33 ± 10.26	0.863
PSA	47	40.43 ± 9.77	34.96 ± 9.91	−5.47 ± 8.02	
Fluency letters					
LA	21	38.81 ± 8.82	32.19 ± 12.93	−6.62 ± 10.31	0.503
PSA	46	35.30 ± 12.83	30.79 ± 13.25	−4.50 ± 9.98	
RAVLT learning total					
LA	20	46.25 ± 10.62	46.95 ± 9.57	0.70 ± 9.14	0.805
PSA	44	43.84 ± 10.01	43.86 ± 11.58	0.02 ± 8.38	
RAVLT recall					
LA	20	9.45 ± 3.41	8.75 ± 2.65	−0.70 ± 2.72	0.603
PSA	44	8.84 ± 2.73	8.50 ± 3.20	−0.34 ± 2.26	
TMT-B					
LA	19	90.74 ± 30.08	104.53 ± 63.80	8.83 ± 50.71	0.092
PSA	44	98.02 ± 52.56	121.12 ± 71.27	23.76 ± 43.64	

Means and standard deviation based on Mann–Whitney U Test. RAVLT recall: Rey’s Auditory verbal learning test recall, RAVLT total: Rey’s Auditory verbal learning test total, TMT-B: Trail Making Test part B, LA: local anesthesia, PSA: procedural sedation and/or analgesia.

## Data Availability

The data presented in this study are available on request from the corresponding author.
